# A draft sequence reference of the *Psilocybe cubensis* genome

**DOI:** 10.12688/f1000research.51613.1

**Published:** 2021-04-09

**Authors:** Kevin McKernan, Liam T. Kane, Seth Crawford, Chen-Shan Chin, Aaron Trippe, Stephen McLaughlin

**Affiliations:** 1 R&D, Medicinal Genomics, Beverly, Mass, 01915, USA; 2 R&D, Oregon CBD, Corvallis, Oregon, 97330, USA; 3 Bioinformatics, DNANexus, Mountain View, CA, 94040, USA

**Keywords:** Psilocybe cubensis, Genome, Single molecule sequencing, Psilocybin

## Abstract

We describe the use of high-fidelity single molecule sequencing to assemble the genome of the psychoactive *Psilocybe cubensis* mushroom. The genome is 46.6Mb, 46% GC, and in 32 contigs with an N50 of 3.3Mb. The BUSCO completeness scores are 97.6% with 1.2% duplicates. The Psilocybin synthesis cluster exists in a single 3.2Mb contig. The dataset is available from NCBI BioProject with accessions PRJNA687911 and PRJNA700437.

## Introduction

There are several mushrooms capable of synthesizing the psychoactive compound psilocybin. This compound has been classified as a “breakthrough therapy” for depression by the FDA ( Johnson and Griffiths 2017). The psilocybin pathway was identified by Fricke et al., but to date no public references exist in NCBI with N50s longer than 50kb ( Fricke et al. 2017; Blei et al. 2018; Fricke et al. 2019a; Fricke et al. 2019b; Blei et al. 2020; Demmler et al. 2020; Fricke et al. 2020). A more contiguous genome assembly can assist in further resolution of the genetic diversity in the fungi’s secondary metabolite production.

## Methods

### DNA isolation

Dried stems from *Psilocybe cubensis* strain P.envy (strain name is anecdotal and obtained in Somerville, MA, USA) were used for isolation of high molecular weight (HMW) DNA using a modified CTAB/Chloroform and SPRI protocol. Briefly, 300mg of stem sample were ground to a fine powder using a -80C frozen mortar and pestle. 150 mg of powder was then aliquoted into 2 mL conical tubes (USA Scientific) with 1.5 mL cetrimonium bromide. These tubes were then incubated at room temperature on a tube rotator for 10 minutes. 6 uL of RNase A (Promega 4 mg/mL) was then added and both tubes were incubated at 37°C for one hour, vortexing every 15 minutes. Following this incubation, 7.5 uL Proteinase K (New England Biolabs 20 mg/mL) was added and the tubes were incubated at 60°C for 30 minutes, vortexing every 10 minutes. At the conclusion of the Proteinase K incubation, both tubes were incubated on ice for 10 minutes. The samples were then centrifuged for 5 minutes at 14000 rpm. 600 uL of supernatant was removed from each tube and added to 600 uL chloroform. The tubes were then vortexed until opaque and spun for 5 minutes at 14000 rpm. 400 uL of the aqueous layer was removed using a wide bore tip and added to a 1.5 mL Eppendorf tube. 400 uL MIP (marijuana infused products) Solution B and 400 uL DNA Binding Beads (Medicinal Genomics PN 420004) were added to the Eppendorf tube and inverted to homogenize. The tubes were then incubated at room temperature on the tube rotator for 15 minutes. The tubes were then removed from the rotator and placed on a magnetic tube rack for 3 minutes. The supernatant was removed, the beads were washed twice with 1 mL of 70% ethanol and allowed to dry for 5 minutes. The beads were then eluted in 100 uL of 56°C Elution Buffer (Medicinal Genomics PN 420004) using a wide bore tip and incubated at 56°C for 5 minutes. Following this incubation, the tubes were returned to the magnetic rack, the supernatant of both tubes were removed using a wide bore tip and pooled in a fresh Eppendorf tube. HMW DNA length was quantified on an Agilent TapeStation and produced a DIN of 8.1. Qubit Fluorometer (Thermo Fisher Scientific) quantified 55ng/ul. Nanodrop Spectrophotometer (Thermo Fisher Scientific) quantified 104ng/ul with 260/280nm ratio of 1.85 and 260/230nm of 0.95.

### Sequencing

Sequencing libraries were constructed according to the manufacturer’s instructions for Pacific Biosciences Sequel II HiFi sequencing. 773,735 CCS reads were generated. Quast ( Gurevich et al. 2013) was used to assess the quality of the input fasta sequence file (N50 = 13.9Kb) and the output assembly fasta file (3.33Mb N50).

### Assembly and annotation

**Figure 1.**
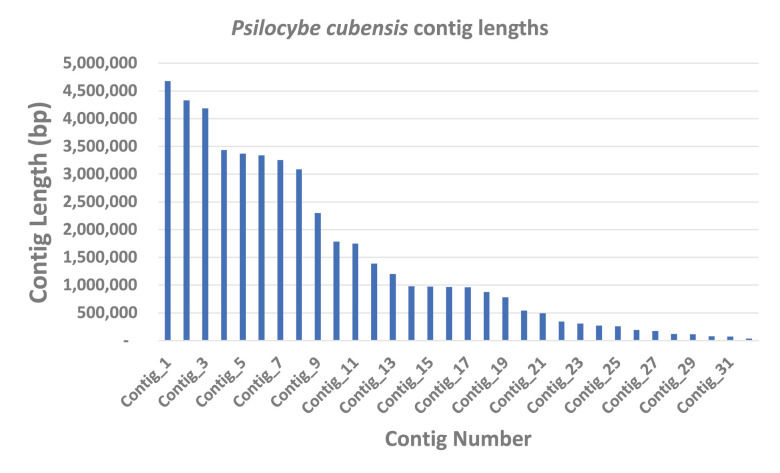
Psilocybe cubensis P.envy contig length distribution (n=32).

The unfiltered CCS data was assembled using the Peregrine assembler (pg_asm 0.3.5,arm_config5e69f3d+) ( Chin 2019). Reads were assembled into 32 contigs with lengths ranging from 32 kilobases to 4.6 megabases ( Figure 1). BUSCO v3.0.2 completeness scores (97.6%) were measured using agaricales_odb10.2020-08-05 BUSCO lineage database ( Table 1) ( Simao et al. 2015; Waterhouse et al. 2018). FunAnnotate v1.8.4 was used to annotate the genome ( Li and Wang 2021) resulting in 13,478 genes.

**Table 1.** BUSCO completeness scores using agaricales_odb10.2020-08-05.

Total BUSCOs	Single-copy	Duplicated	Fragmented	Missing
3870	3729	45	9	87
97.60%	96.40%	1.20%	0.20%	2.20%

The final genome assembly was aligned to three other public *Psilocybe cubensis* datasets ( Fricke et al. 2017; Torrens-Spence et al. 2018; Reynolds et al. 2018) and one different *Psilocybe* species ( *Psilocybe cyanescens)* to verify taxonomic identification ( Table 2). In total, 96-98.75% of these *Psilocybe cubensis* sequences align to the new HiFi generated *Psilocybe cubensis* P.envy reference using minimap2 and bwa-mem ( Li and Durbin 2010; Li 2018). Mapping rates were determined using samtools flagstat on bam files ( Li et al. 2009). Alignments were visualized with MUMmer V4.0.0beta2 and Integrative Genomics Viewer v2.4.16 ( Delcher et al. 2003; Robinson et al. 2011; Thorvaldsdottir et al. 2013).

**Table 2.** Three *Psilocybe cubensis* data sets in NCBI and JGI were aligned to the P.envy HiFi reference. A different Psilocybe species ( *Psilocybe cyanescens*) genome was also mapped with much lower mapping efficiency.

Author	Accession	Data type	Mapping rate	Tool	Species
Fricke et al. 2017	https://mycocosm.jgi.doe.gov/Psicub1_1/Psicub1_1.home.html	Illumina Assembly	98.8%	Minimap2	*P. cubensis*
McKernan et al. 2020	NCBI Project: PRJNA687911	Illumina FastQ	96.0%	bwa-mem	*P. cubensis*
Torrens-Spence et al. 2018	NCBI Project: PRJNA450675	RAN-Seq Assembly	98.5%	Minimap2	*P. cubensis*
Reynolds et al. 2018	NCBI Project: PRJNA387735	Illumina Assembly	56.8%	Minimap2	*P. cyanescens*

**Figure 2.**
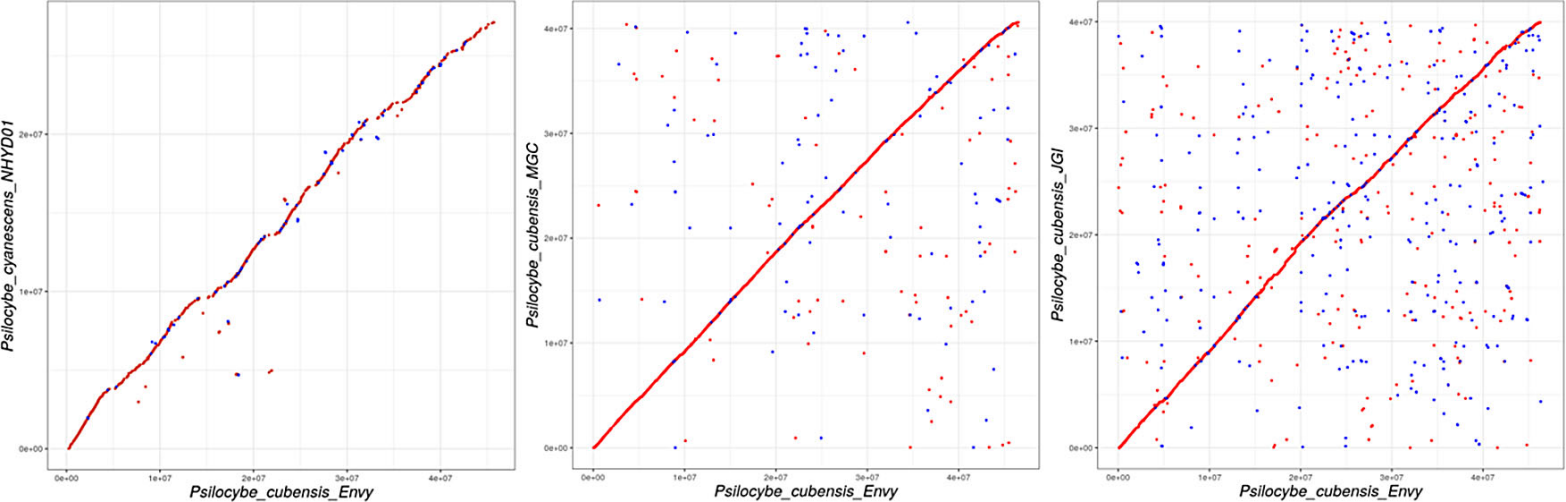
Whole genome alignments of short read Illumina assemblies to *Psilocybe cubensis* strain P. envy. Left is *Psilocybe cyanescens* from Reynolds et al. Middle is McKernan et al. (MGC) Illumina assembly. Right is Fricke et al. or JGI assembly.

Three Illumina genome assemblies (Reynolds et al., McKernan et al., Fricke et al.) were additionally aligned using MUMmer for whole genome alignment plots ( Figure 2).

### Polymorphisms

183,430 SNPs were identified with GATK v4.1.6.0 by comparing Illumina whole genome shotgun data (McKernan et al. NCBI Project: PRJNA687911) with the HiFi reference assembly. This equates to a SNP every 243 bases.

### Structural variation

**Figure 3.**
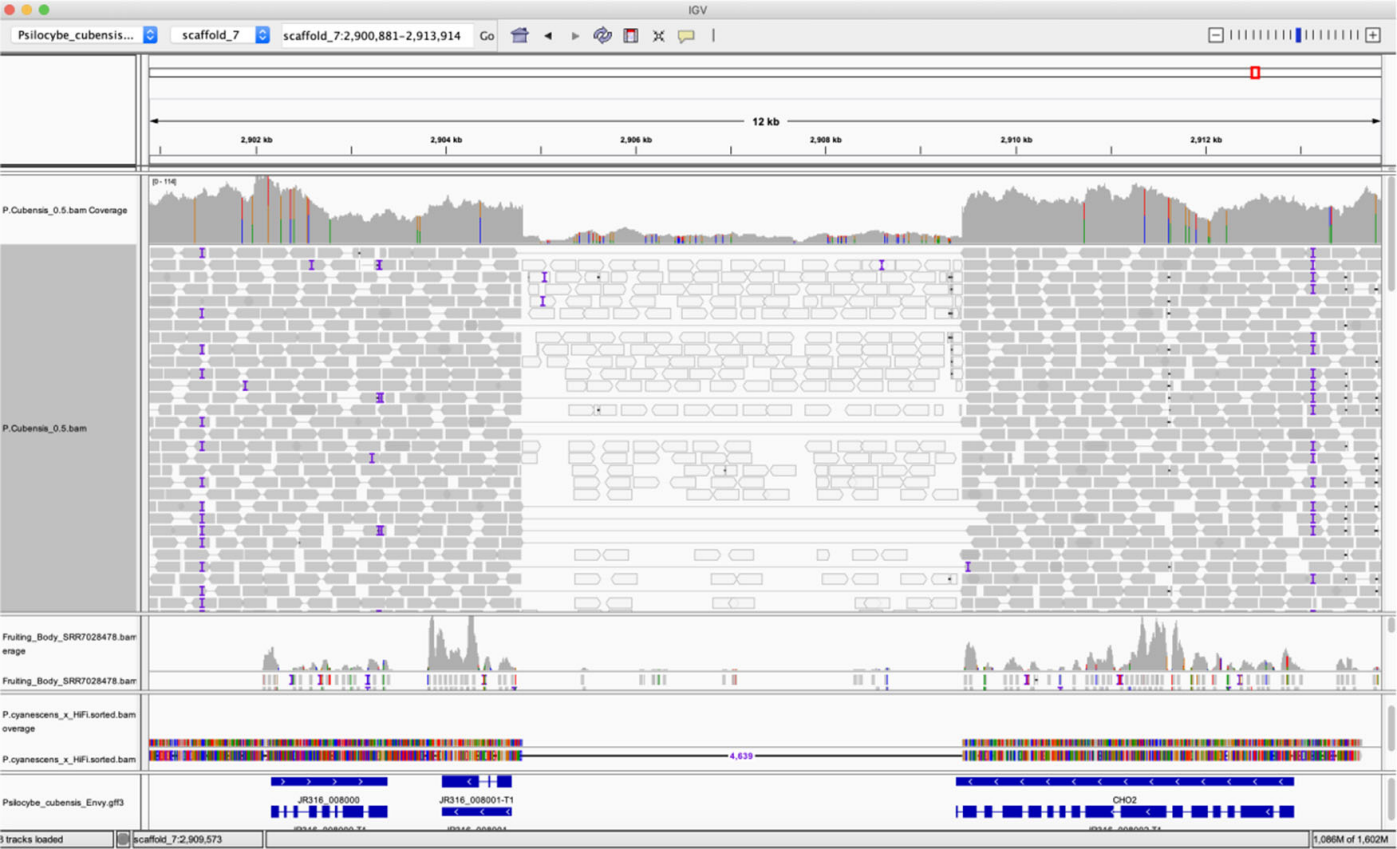
IGV display of Illumina reads mapped to HiFi *Psilocybe cubensis* P.envy assembly. Top track is Medicinal Genomics Illumina whole genome shotgun data of a different *P. cubensis* (strain name unknown: NCBI Project: PRJNA687911) mapped to the HiFi *P. cubensis* strain P.envy. Second track contains RNA-Seq data from a third *P. cubensis* genome (strain name also unknown: NCBI Project: PRJNA450675) hosted at JGI. Third track is *Psilocybe cyanescens* genome mapped to HiFi *P. cubensis* P.envy reference genome. Fourth track is FunAnnotate GFF3 annotation of the HiFi *P. cubensis* P.envy genome.

The N-methyltransferase gene responsible for Psilocybin production in P.envy contains a structural variation not seen in previous *P. cubensis* surveys ( Figure 3). Illumina read mapping of the McKernan et al. *P. cubensis* assembly in NCBI (NCBI Project: PRJNA687911) demonstrates multiple read pairs spanning a 4.6kb insertion in the HiFi *P. cubensis* strain P.envy. This insertion extends the 3’ end of the P.envy N-methyltransferase gene. The 4.6kb insertion is also observed as a deletion in *Psilocybe cyanescens* and as a deletion in RNA-Seq data from Torrens-Spence et al. (NCBI Project: PRJNA450675) ( Reynolds et al. 2018). Further work is required to understand the biological significance of this variation.

## Conclusions

A highly contiguous *Psilocybe cubensis* genome has been generated. The N50 contigs lengths are 75 fold more contiguous than the existing assembly available at JGI. This reference can aid in the identification of genetic variation that may impact psilocybin, psilocin, norpsilocin, baeocystin, norbaeocystin and aeruginascin production.

## Data availability

GenBank: Psilocybe cubensis strain MGC-MH-2018, whole genome shotgun sequencing project, Accession number JAFIQS000000000.1: https://www.ncbi.nlm.nih.gov/nuccore/JAFIQS000000000.1/.

BioProject: Psilocybe cubensis, Accession number PRJNA687911: https://www.ncbi.nlm.nih.gov/bioproject/PRJNA687911

BioProject: Psilocybe cubensis strain: MGC-MH-2018, Accession number PRJNA700437: https://www.ncbi.nlm.nih.gov/bioproject/PRJNA700437

CoGe genome browser: Psilocybe cubensis (Psilocybe cubensis P.envy), https://genomevolution.org/coge/GenomeInfo.pl?gid=60487
