# Supplementary material for: A draft reference assembly of the Psilocybe cubensis genome
Source: F1000Res. 2021 Jun 15;10:281. Originally published 2021 Apr 9. [Version 2] doi: 10.12688/f1000research.51613.2 (PMC8220353; doi:10.12688/f1000research.51613.2)
Supplement: Peer review history

# Peer review: Reviewer response for version 2

**DOI:** 10.5256/f1000research.57314.r87516

**Published:** 2021-06-22

**Authors:** Jason Slot

**Affiliations:** 1 Department of Plant Pathology, The Ohio State University, Columbus, OH, USA

The authors have provided changes for all the comments and requested edits and I deem all of them to be acceptable, and to have much improved the manuscript. There remains one typo that the authors may wish to correct: In the last section of the Polymorphisms section, "utilized for" should replace "utilized to for".

Are sufficient details of methods and materials provided to allow replication by others?

Partly

Is the rationale for creating the dataset(s) clearly described?

Yes

Are the datasets clearly presented in a useable and accessible format?

Partly

Are the protocols appropriate and is the work technically sound?

Yes

Reviewer Expertise:

Fungal ecology, microbial genomics, evolution, metabolism

I confirm that I have read this submission and believe that I have an appropriate level of expertise to confirm that it is of an acceptable scientific standard.

# Peer review: Reviewer response for version 1

**DOI:** 10.5256/f1000research.54802.r83076

**Published:** 2021-05-21

**Authors:** Philippe Henry, Anders Goncalves da Silva

**Affiliations:** 1 Lighthouse Genomics Inc., British Columbia, Canada; 2 Egret Bioscience Ltd., West Kelowna, Canada; 3 Lighthouse Genomics Inc., BC, Canada

McKernan and colleagues present on the first highly contiguous draft genome for the magic mushroom *Psilocybe cubensis*. We commend their use of High accuracy long read sequencing and an advanced bioinformatics pipeline to build a much more complete picture of the *P. cubensis* genome and for making it openly available to the public with the promise the genetic architecture of tryptamine expression in magic mushrooms. 

 The methods employed are state of the art and the authors provide sufficient access to the data to enable peers and the public to replicate the experiment. While they acknowledge that the HiFi sequencing approach comes with great advantages, in particular greatly improved contiguity and and BUSCO completeness scores compared to other *P. cubensis* genomes published to date, the authors did not acknowledge that fungi can have multiple nuclei in a cell, sometimes with completely different haplotypes. As such, we posit that their assembly could possibly be a metagenome assembly, rather than the assembly of a single genome, thus providing an alternative explanation to the large insertion detected in the norbaeocystin methyltranferase (psiM) gene.

 Perhaps a means to provide a remedy to this is to provide some additional background on the *P. cubensis* Penis Envy (PE) strain, in particular the alleged origin of the PE mutant and its probable clonal propagation. While anecdotal at best, the fabled “mutation” of PE appeared and was selected from a phenotype of an amazonian *P. cubensis* accession, as a towering fruiting body with a pale cap and missing partial veil, which was then preserved via clonal propagation. The mutant is also described as being more potent that most other *P. cubensis* strain, leading to the hypothesis that it had a skewed drug to prodrug ratio (psilocyn/psilocybin) which would hint to a mutation in the psiK gene as opposed to the large insertion in psiM.

 Other putative mechanisms could be polymorphisms at other loci involved in the psilocybin biosynthetic pathways as well as ancillary genes involved in the SAM salvage pathway (e.g. ref 1), a list of putative functional SNPs that may interact with the large insertion is shown here from an earlier version of the *P.cubensis* genome. Genotyping several strains at the 4.6kb insertion and ancillary SNPs may help shed light on the mechanism behind the higher perceived potency of PE compared to other *P. cubensis* strains and other species in the *Psilocybe *and *Panaeolus* genus, In that vein, the authors may gain additional insight by including the *P. serbica var bohemica *genome to their comparative analysis, provided that chemotypic information associated with each accessions is made available.

 Node Position Target gene Ontogeny

 NODE_599 19295 sahH *S‐adenosyl‐l‐homocysteine hydrolase*

 NODE_599 19304 sahH *S‐adenosyl‐l‐homocysteine hydrolase*

 NODE_599 19415 sahH *S‐adenosyl‐l‐homocysteine hydrolase*

 NODE_599 19421 sahH *S‐adenosyl‐l‐homocysteine hydrolase*

 NODE_599 19744 sahH *S‐adenosyl‐l‐homocysteine hydrolase*

 NODE_599 19746 sahH *S‐adenosyl‐l‐homocysteine hydrolase*

 NODE_599 20318 sahH *S‐adenosyl‐l‐homocysteine hydrolase*

 NODE_599 20779 sahH *S‐adenosyl‐l‐homocysteine hydrolase*

 NODE_599 20801 sahH *S‐adenosyl‐l‐homocysteine hydrolase*

 NODE_599 20840 sahH *S‐adenosyl‐l‐homocysteine hydrolase*

 NODE_6392 313322 samS *S‐adenosyl‐l‐methionine synthetase*

 NODE_6392 314122 samS *S‐adenosyl‐l‐methionine synthetase*

 NODE_6392 314300 samS *S‐adenosyl‐l‐methionine synthetase*

 NODE_712 1234504 metS l‐methionine synthetase

 NODE_755 63920 psiD tryptophan decarboxylase 

 NODE_755 64575 psiD tryptophan decarboxylase 

 NODE_755 64576 psiD tryptophan decarboxylase 

 NODE_755 65088 psiD tryptophan decarboxylase 

 NODE_755 65181 psiD tryptophan decarboxylase 

 NODE_755 61250 psiM methyltransferase

 NODE_755 61711 psiM methyltransferase

 NODE_755 62335 psiM methyltransferase

 NODE_755 58208 psiT2 major-facilitator-type transporters 

 NODE_755 58407 psiT2 major-facilitator-type transporters 

 NODE_755 58772 psiT2 major-facilitator-type transporters 

 NODE_755 58883 psiT2 major-facilitator-type transporters 

 NODE_755 59054 psiT2 major-facilitator-type transporters 

 NODE_755 59321 psiT2 major-facilitator-type transporters 

 NODE_755 59372 psiT2 major-facilitator-type transporters 

 NODE_755 59426 psiT2 major-facilitator-type transporters 

 NODE_755 59484 psiT2 major-facilitator-type transporters 

 NODE_755 59492 psiT2 major-facilitator-type transporters 

 NODE_755 59504 psiT2 major-facilitator-type transporters 

 NODE_755 59522 psiT2 major-facilitator-type transporters 

 NODE_755 59537 psiT2 major-facilitator-type transporters 

 NODE_755 59540 psiT2 major-facilitator-type transporters 

 NODE_755 59566 psiT2 major-facilitator-type transporters 

 NODE_755 59594 psiT2 major-facilitator-type transporters 

 NODE_755 59648 psiT2 major-facilitator-type transporters 

 NODE_755 59694 psiT2 major-facilitator-type transporters 

 NODE_755 59771 psiT2 major-facilitator-type transporters 

 NODE_755 59869 psiT2 major-facilitator-type transporters 

 NODE_755 59875 psiT2 major-facilitator-type transporters 

 NODE_755 56543 psiH P450 monooxygenase 

 NODE_755 54126 psiK Kinase

 NODE_755 54132 psiK Kinase

 NODE_755 54136 psiK Kinase

 NODE_755 54159 psiK Kinase

 NODE_755 54213 psiK Kinase

 NODE_755 54223 psiK Kinase

 NODE_755 54311 psiK Kinase

 NODE_755 51932 psiT1 major-facilitator-type transporters 

 NODE_755 52391 psiT1 major-facilitator-type transporters 

 NODE_755 52473 psiT1 major-facilitator-type transporters 

 NODE_755 52477 psiT1 major-facilitator-type transporters 

 NODE_755 52491 psiT1 major-facilitator-type transporters 

 NODE_755 52620 psiT1 major-facilitator-type transporters 

 NODE_755 52688 psiT1 major-facilitator-type transporters 

 NODE_755 52787 psiT1 major-facilitator-type transporters 

 NODE_755 52793 psiT1 major-facilitator-type transporters

Are sufficient details of methods and materials provided to allow replication by others?

Yes

Is the rationale for creating the dataset(s) clearly described?

Yes

Are the datasets clearly presented in a useable and accessible format?

Yes

Are the protocols appropriate and is the work technically sound?

Yes

Reviewer Expertise:

Population genetics, genotype-chemotype

We confirm that we have read this submission and believe that we have an appropriate level of expertise to confirm that it is of an acceptable scientific standard.

## References

[1] : S ‐Adenosyl‐l ‐Methionine Salvage Impacts Psilocybin Formation in “Magic” Mushrooms. *ChemBioChem* .2020;21(9) : 10.1002/cbic.201900649 1364-1371 10.1002/cbic.201900649 PMC731753131802575

# Peer review: Reviewer response for version 1

**DOI:** 10.5256/f1000research.54802.r83396

**Published:** 2021-04-26

**Authors:** Jason Slot

**Affiliations:** 1 Department of Plant Pathology, The Ohio State University, Columbus, OH, USA

**Summary:**

 The manuscript presents a high quality assembly of the historically and medicinally important fungus, *Psilocybe cubensis*. Best practices were observed in sequencing, assembly, and annotation. The manuscript notes a potentially important structural variation present in the *P. envy* strain psilocybin N-methyltransferase gene, which resembles the variation in the more potent, by psilocybin content, *Psilocybe cyanescens*.

- **Is the rationale for creating the dataset(s) clearly described?**

The study was undertaken in order to provide a high quality reference genome for the species. To date, the genomes in the species and genus are fragmented more than is desirable for basic and applied comparative investigations of genome content and architecture.

- **Are the protocols appropriate and is the work technically sound?**

The Pacific Biosciences Sequel II HiFi methods used for sequencing are among the best for generating near-chromosome level assembly. Assembly was performed with cutting-edge Peregrine Assembler, and the annotation appropriately used the fungus-specific FunAnnotate pipeline. Single nucleotide polymorphisms (SNP) were called with appropriate software, but parameters were not detailed in the text. This and structural variation were not intended to be exhaustive, but provide intriguing statistics and examples to warrant follow-up investigations.

- **Are sufficient details of methods and materials provided to allow replication by others?**

Parameters for SNP calling would have to be further detailed in order to allow replication of raw SNP numbers between two isolates.

- **Are the datasets clearly presented in a useable and accessible format?**

Figure 1 and both tables are clear and informative. Figure 2 readability would be improved by increasing the size of the axis titles. Figure 3 is not sufficiently informative or simple to acquire meaning as it is currently presented. This figure would benefit from marking the "tracks" clearly, but number and perhaps with additional labels for RNAseq, *P. cyanescens*, and annotation as the IGV display is too small to read as is. Is there significance to the locus that is presented in Figure 3? If not, then perhaps indicate it is a "representative" locus.

**Other comments:**

- The title might flow better as "A draft reference assembly of...".
- In the introduction, "several mushrooms" might better be stated "about 200 mushroom species".
- In the Introduction " fungi's " would be more syntactically correct as "fungus' "
- Given that dried stems were used for genomic DNA isolation, it is expected that some additional microbial DNA might be present. Authors should note if the mushroom was produced axenically, or if contaminant reads were filtered to either prevent or address presence of additional species' genomes in the assembly.
- Citations are incomplete in the last sentence of "Assembly and annotation" section, and second sentence of "Structural variation" section.

Are sufficient details of methods and materials provided to allow replication by others?

Partly

Is the rationale for creating the dataset(s) clearly described?

Yes

Are the datasets clearly presented in a useable and accessible format?

Partly

Are the protocols appropriate and is the work technically sound?

Yes

Reviewer Expertise:

Fungal ecology, microbial genomics, evolution, metabolism

I confirm that I have read this submission and believe that I have an appropriate level of expertise to confirm that it is of an acceptable scientific standard, however I have significant reservations, as outlined above.

# Response

**Published:** 2021-05-21

**Authors:** Kevin McKernan

**Affiliations:** Medicinal Genomics Corporation, USA

The reviewer makes excellent points. We will be making these suggested changes to the final manuscript.
